# Detection and Classification of Histopathological Breast Images Using a Fusion of CNN Frameworks

**DOI:** 10.3390/diagnostics13101700

**Published:** 2023-05-11

**Authors:** Ahsan Rafiq, Alexander Chursin, Wejdan Awad Alrefaei, Tahani Rashed Alsenani, Ghadah Aldehim, Nagwan Abdel Samee, Leila Jamel Menzli

**Affiliations:** 1 School of Automation, Chongqing University of Posts and Telecommunications, Chongqing 400065, China; l201710003@stu.cqupt.edu.cn; 2 Higher School of Industrial Policy and Entrepreneurship, RUDN University, 6 Miklukho-Maklaya St, Moscow 117198, Russia; chursin-aa@rudn.ru; 3 Department of Programming and Computer Sciences, Applied College in Al-Kharj, Prince Sattam Bin Abdulaziz University, Al-Kharj 16245, Saudi Arabia; wejdan.awad.1421@gmail.com; 4 Department of Biology, College of Sciences in Yanbu, Taibah University, Yanbu 46522, Saudi Arabia; thn1233r@gmail.com; 5 Department of Information Systems, College of Computer and Information Sciences, Princess Nourah bint Abdulrahman University, P.O. Box 84428, Riyadh 11671, Saudi Arabia; lmjamel@pnu.edu.sa; 6 Department of Information Technology, College of Computer and Information Sciences, Princess Nourah bint Abdulrahman University, P.O. Box 84428, Riyadh 11671, Saudi Arabia; nmabdelsamee@pnu.edu.sa

**Keywords:** breast cancer, histopathological images, deep learning, machine learning, convolutional neural network

## Abstract

Breast cancer is responsible for the deaths of thousands of women each year. The diagnosis of breast cancer (BC) frequently makes the use of several imaging techniques. On the other hand, incorrect identification might occasionally result in unnecessary therapy and diagnosis. Therefore, the accurate identification of breast cancer can save a significant number of patients from undergoing unnecessary surgery and biopsy procedures. As a result of recent developments in the field, the performance of deep learning systems used for medical image processing has showed significant benefits. Deep learning (DL) models have found widespread use for the aim of extracting important features from histopathologic BC images. This has helped to improve the classification performance and has assisted in the automation of the process. In recent times, both convolutional neural networks (CNNs) and hybrid models of deep learning-based approaches have demonstrated impressive performance. In this research, three different types of CNN models are proposed: a straightforward CNN model (1-CNN), a fusion CNN model (2-CNN), and a three CNN model (3-CNN). The findings of the experiment demonstrate that the techniques based on the 3-CNN algorithm performed the best in terms of accuracy (90.10%), recall (89.90%), precision (89.80%), and f1-Score (89.90%). In conclusion, the CNN-based approaches that have been developed are contrasted with more modern machine learning and deep learning models. The application of CNN-based methods has resulted in a significant increase in the accuracy of the BC classification.

## 1. Introduction

Mammary cancer, commonly known as breast cancer (BC), is a form of life-threatening cancer that primarily affects women. When compared to other forms of cancer, the mortality rate associated with BC for female patients is the second highest [1]. Breast cancer is a collection of cells that begin in a woman’s breast and have the ability to rapidly spread to any other organ in the body [2]. These cells have grown in an uncontrolled manner. There are many subtypes of cancer, but the cancers that most commonly strike humans are lung cancer, breast cancer, and skin cancer. The World Health Organization (WHO) mentioned that the mortality ratio from cancer is up to 7.2 million for lung cancer, 1.3 million fatalities for skin cancer, and 512,030 deaths from breast cancer [3,4]. This statistic refers to the number of people who have died from lung cancer. Breast cancer patients who have tumors that are even less than 10 mm in size have a 98% chance of surviving the disease [5]. Because of this, the likelihood of successfully surviving breast cancer is strongly connected with the volume of the tumor. The extent of a breast tumor is one of the primary factors that determines whether a patient will make it through their cancer treatment. There are different ways of thinking about BC that can be taken into consideration in order to recognize and categorize it. Imaging techniques such as ultrasound [6], X-rays [7], digital mammograms [8,9,10,11,12,13,14], and CT scans [15,16] are more useful. Researchers are able to identify the many types of cancer utilizing a number of techniques, such as early-phase screening (EPS), etc. In addition to this, they have developed original strategies for the diagnosis and treatment of cancer at earlier stages, which has improved patient outcomes. Large volumes of information relating to cancer have been accumulated as a result of the development of breakthrough technologies in medicine, and this information has been made available in the field of bioinformatics as well as the scientific community for the purposes of investigation and analysis. Predicting whether a patient will get breast cancer is, nevertheless, one of the most challenging and tough tasks in the healthcare profession. This includes the danger of incorrect classification when utilizing the diagnostic procedures that have been investigated previously. In addition, a diagnosis of breast cancer in a clinic is dependent on the visual examination of patients by pathologists, who are highly trained specialists in the field of cancer. Pathologists study cancer extensively and have extensive clinical experience. On the other hand, this procedure is performed manually, which is not only labor-intensive but also time-consuming and prone to human mistakes. This is because the vast majority of cells have arbitrary shapes, differing portions, and uncontrolled visual angles. This is the reason why this occurs. Before beginning early therapy, it is critical to determine if a tumor is cancerous (also known as malignant) or non-cancerous (also known as benign).

The use of machine learning (ML) [17,18,19,20] and deep learning (DL) [21,22] methodologies for the automatic categorization of BC has assisted in decreasing the risk of getting cancer and recurrence, and survival prediction might increase the accuracy by 20% to 25% more than it did in the previous year [23]. In the identification of invasive breast cancer, ML/DL is a technique that sees widespread application [8,9,10,11,12,13,14,24]. The results that have been obtained through DL-based models have been superior to those that have been obtained through the more conventional approaches to machine learning [10]. In most cases, the application of DL approaches [21,22,25,26,27,28] is founded on the use of an only one deep learning algorithm including RNN, LSTM, or CNN. Even though these DL-based models have obtained good performance, the employment of hybrid deep learning models [29,30,31,32,33,34,35,36,37,38] has assisted in improving the achieved classification performance. This was possible owing to the assistance provided by hybrid deep learning models. Because of this, we decided to propose a fusion-based CNN model to categorize breast cancer histology.

The following is a list of the most important contributions that may be summarized as follows:

- A fusion-based CNN model is presented here for the purpose of classifying the histology of breast cancer.
- The presented models are applied in clinical and biomedical research with the goal of developing a successful identification method for breast cancer in breast tissue.
- The models that have been built are predicated respectively on the convolutional neural network (CNN) model, the fusion of two CNNs (often referred to as 2-CNN), and three CNNs (3-CNN).

When compared to state-of-the-art approaches, the proposed model performed significantly better when classifying breast cancer histology. The suggested 3-CNN model’s accuracy has outperformed, by a percentage point of 30%, that of the conventional ML approaches, ANN, for the binary classification (benign/malignant) of breast tissue. While the current CNN-based model for the binary classification of breast tissues is quite accurate, the suggested 3-CNN model introduces an improvement, 1%, in the model’s accuracy. The paper is arranged as follows: the first section includes the experimental dataset, and its preprocessing. In Section 2, we explore the various techniques utilized using CNN-based fusion models. In Section 3, we offer the experimental study of the recommended methodologies, which covers ML and DL approaches, and in Section 4, we state the results obtained and we discuss the findings. In Section 5, the conclusion and recommendations for additional research are given.

### Related Work

Despite the numerous research that has been undertaken and published, as well as the substantial breakthroughs in the automatic identification of BC features, a number of issues have led to major difficulties in image categorization [39]. These factors include the inconsistency in tissue and cell shape, the phenomenon of cell overlapping, the visual heterogeneity of stained histopathological analysis, and inconsistent color distribution. Despite these factors, significant advances have been made in the automatic detection of BC characteristics [39]. These issues pose significant challenges in the accurate automatic detection of BC [40]. The problem is due to high resolution of pathologist images (PI), making it difficult to automatically transplant some of the effective techniques from natural images to PI. The majority of the early classification methods for BC-PI were based on a binary system that divided BC lesions into two categories: cancerous and non-cancerous [10,41,42,43,44,45] or a more sophisticated tri-classification as invasive carcinoma, situ carcinoma, and normal [46,47]. Most of the work was done using whole images or on extracted nuclei relying on architectural, morphological, and textural aspects using the standard ML approach. It is in-depth mentioning that most of the classification methods were performed on small resolution images at various magnifications. Moreover, these approaches used artificial-rely on the extraction of feature methods, which not only require a significant amount of effort and professional domain expertise but also face certain problems in extracting distinguishing high-quality features. Consequently, employing traditional ML approaches is severely limited when it comes to the classification of BC. Finally, DL algorithms [17,48] achieved impressive success of wide range of biomedical applications [11,12,13,14].

The ML/DL method is one that is utilized frequently in the process of determining whether a patient has breast cancer [8,9,10,11,12,13,14,24]. The results that have been acquired through the use of DL-based models have been demonstrated to be superior to those that have been produced through the use of more conventional approaches to machine learning [10]. The application of DL techniques in healthcare [21,22,25,26,27,28] is typically predicated on the deployment of a unique deep learning model such as CNN, long short-term memory (LSTM), or recurrent neural network (RNN). This is the case in the vast majority of the situations. Despite the fact that these DL-based models have achieved good performance, the utilization of hybrid deep learning models [29,30,31,32,33,34,35,36,37,38] has contributed to the improvement of the classification performance that was accomplished.

Recently, a small group of researchers has argued that AI should be used to automatically detect and diagnose histopathological abnormalities in breast lesions. Recent studies have employed ML/DL algorithms to detect breast tumors; Table 1 highlights the current status of the field and the shortcomings of each technique. The most noteworthy aspects of the new system are highlighted in the table.

Among DL techniques, the CNN is the most important. CNNs are widely used in the classification of BC. Bayramoglu et al. [48] employed the magnification DL algorithm used BreaKHis dataset as well, with an 83% accuracy rate. Arajo et al. [49] conducted an initial investigation on multiple classifications for BC-PI. They were able to recover features by employing a CNN that was analogous to AlexNet, and after that, they used SVM to classify the characteristics that were gathered. On the other hand, a recurrent neural network (RNN) is only occasionally used in applications that include the classification of Histology Images (HI). In contrast to the RNN, the CNN is able to process data by using its own internal state. A multitude of remarkable CNN-based algorithms for the automated and precise categorization of BC have been recently developed [50] in preparation for the ICIAR2018 competition. These methodologies have been extremely significant in propelling the state of the art forward. These techniques’ core concepts are similar. After the high-resolution image (HI) has been pre-processed and data-enhanced, they are then split into patches of a similar size, and the features recovered using a CNN are then acquired from each patch individually. Vesal et al. [51] presented a transfer learning (TL) approach. They categorized each patch of one picture using the simple majority approach of ResNet and GoogLeNet. Vang et al. [52] were the first to suggest utilizing Google Inception accomplish patch-wise categorization. In order to arrive at an image-wise prediction, the patch-wise detection was put through an ensemble fusion architecture that includes logistic regression, gradient boosting, and majority voting. Deep convolutional feature representation was suggested by Rakhlin et al. [53]. The features have been extracted using a generic CNN. At the end, for the final classification result, they employed gradient enhanced trees. ResNet was used by Awan et al. [54] to generate 12-dimensional vector features representing twelve non-overlapped patches of 512 * 512. The final image-wise classification result was based on the majority vote result on the categorization of 1024 * 1024 pixel-overlapping blocks of patches.

In comparison to the present state of the art in utilizing machine and deep learning (ML/DL) to classify BC histology, the network demonstrates improved performance in properly and precisely classifying breast tissues, suggesting its increased application in this area. Moreover, it is unclear how the use of hybrid DL models aids in achieving a high degree of precision when classifying BC samples. The foregoing research demonstrates definitively that hybrid DL models outperform their single deep learning (DL) counterparts when it comes to BC detection and classification. To further enhance BCs’ ability to classify data, in view of the aforementioned information, we came up with a complex deep learning approach to classify BCs from HI pictures using a hybrid CNN learning model.

## 2. Material and Methods

This section describes the framework of histopathological BC cancer detection, including data collection and data augmentation methods. Furthermore, this section has been extended including the proposed methodology.

### 2.1. Dataset and Image Augmentation Methods

**Figure 1:**
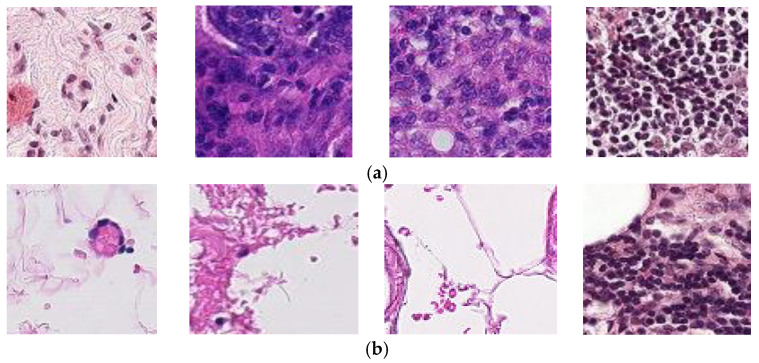
Examples of benign and malignant tissues. (**a**) Examples of Malignant tissues; (**b**) Examples of benign tissues.

The dataset related to BC is obtained from Kaggle [55]. This dataset possesses originally PCam dataset, including no repeated samples. This collection has a total of 22,090 BC-HI with patches extracted from 200 scan slide images. These images were obtained from 162 women who had breast cancer and had it identified and tested at the University Medical Center in the Netherlands. The experimental training dataset, contains 170 WSIs BC-HI samples, and the training dataset includes 100 WSIs, are considered training datasets. The images in this collection have a dimension of 2040 pixels wide by 1536 pixels high. For the purpose of maintaining uniformity, each slide was scanned using the same scanner at a resolution of 0.25 micro/pr. Lower-resolution images were sampled to a more manageable 50 pixels by 50 pixels. Malignant and non-metastatic tumors (benign) are the two primary classes of BC in the Kaggle dataset. It includes a total of 220,025 scan images of BC, consisting of 130,893 non-cancers (benign) and 89,117 images of malignant cases (cancer images). The Kaggle data set is split into a training data set (80%) and a testing data set (20%). To avoid overfitting, the best results may be produced, according to empirical research, if just 20–30% of the data are used for testing, with the rest 70–80% of the data being used for training. Both classes’ samples in the RGB color space and the PNG file format are utilized for the image files and have label 0 for benign and 1 for malignant as shown in Figure 1.

**Figure 2:**
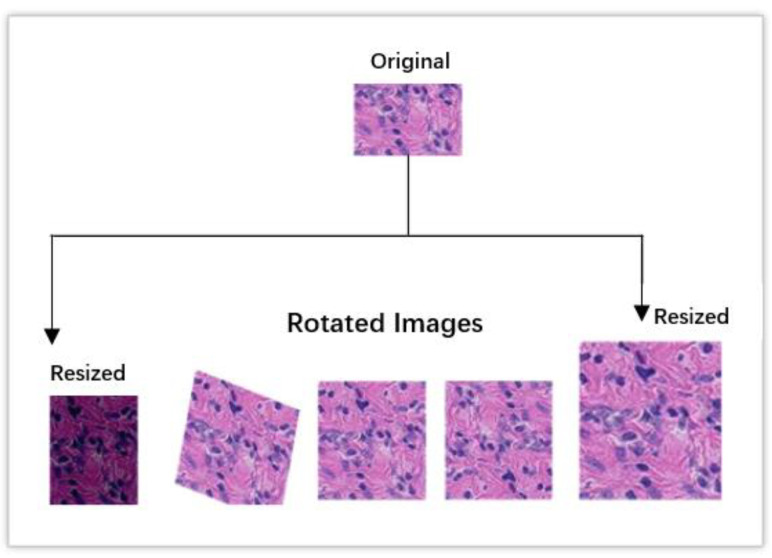
Examples of augmented images via rotation and resizing.

As there were sufficient photos of the benign class in the data set utilized for this study, the augmented method was not applied to these images. The number of images that belonged to the malignant class, which was 89,117, was raised to 900,000. The accuracy of CNN architectures in the classification process can be improved through the application of data augmentation techniques [22,31,34] as shown in Figure 2.

The images that were used in this investigation were rotated in various ways before being examined. In this particular investigation, the rotation rate is set at 25. The original images as well as the augmented versions were used to compile the data set for this investigation. The only time the augmented images were shown was when the model was being trained. In order to prevent overfitting, the test makes use of the original data.

### 2.2. Proposed Model

For detecting and classifying tasks, the CNN based classifier (1-CNN) is adopted. Moreover, we presented further classifiers that fused two CNN models and three CNN models.

A. The 1-CNN model

**Figure 3:**
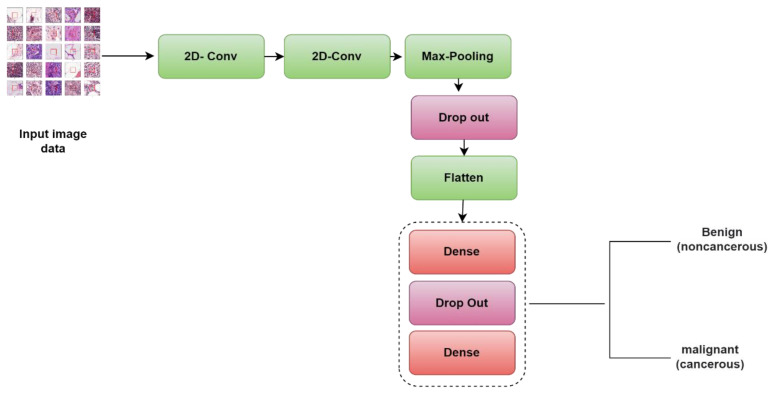
Block diagram of the 1-CNN model for the classification of breast histological images into benign/malignant tissues.

We have developed a basic 1-CNN as a method for distinguishing between malignant and benign cancer as shown in Figure 3. This 1-CNN model is put together using the following components:

- Two convolutional layers, each having 32 filters and a kernel size of (3, 3), with the ReLU serving as the activation function. One of the convolutional layers has 32 filters with a kernel size of (3, 3).
- A maximum pooling layer with the pool size set equal to (2, 2)
- A layer with dropouts refers to the process of ignoring neurons during the training step of a randomly selected neurons set; the rate that we use is 0.25.
- A flattened layer.
- A dense layer where all one-layer inputs are connected to each succeeding layer’s activation functions. The dense layer has 64 levels with the ReLU serving as an activation function.
- Another dropout layer having a rate of 0.5.
- A dropout layer that has an activation function that is sigmoid.
- The number of epochs is a hyperparameter that controls how many times the training process will iterate. When we talk about an epoch, we imply that every single training dataset has been given the chance to bring the parameters of the internal model up to date. In the course of our research, we utilized a total of thirty epochs.

B. The 2-CNN model

For BC classification, we suggest a hybrid approach that combines two CNN models. Combining the results of many CNN architectures, each of which may be beneficial in extracting a certain feature from histopathological images of BC, may allow us to develop general features that are useful in the classification of BC.

**Figure 4:**
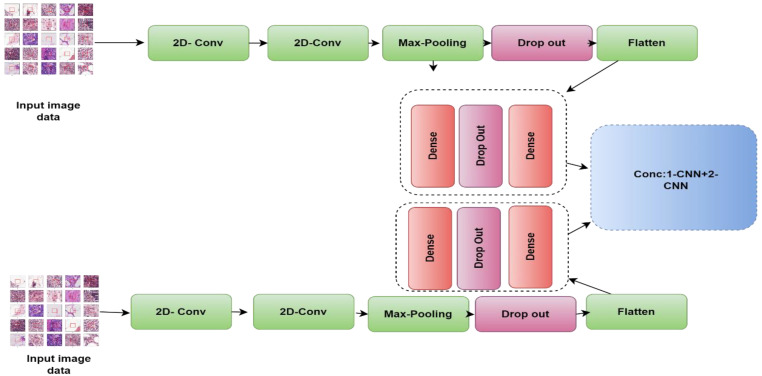
Block diagram of the 2-CNN model for the classification of breast histological images into benign/malignant tissues.

The 2-CNN model is made up of two CNN models that are very close to one another and have the same architecture as the previous model. Figure 4 shows how the 1-CNN and 2-CNN models are combined; i.e., the two models are firstly concatenated; then, a dense layer employing a SoftMax activation function (AF) is included

C. The 3-CNN model

**Figure 5:**
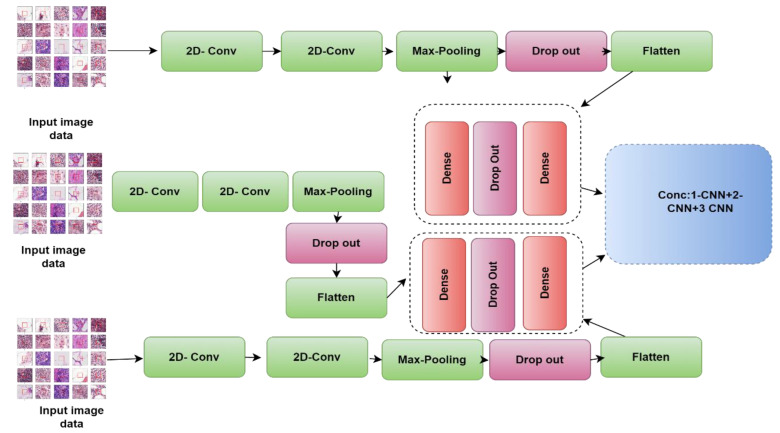
The proposed 3-CNN model block diagram for the classification of breast histological images into benign/malignant tissues.

The 3-CNN model is constructed using three CNN models that are quite close to one another; as shown in Figure 5, we combined the 1-CNN, 2-CNN, and 3-CNN models. In fact, once the first two CNN models are combined, a second combination with the third model is processed. At the end, a dense layer and a SoftMax AF are added.

D. Optimizer

We use an optimizer through its paces in order to decrease the amount of error in the predictions (the loss function), as well as to improve the accuracy of the predictions and get them as close to optimal as possible. We employed an optimizer known as ADAM (Adaptive Moment Estimation), which is based on the first-order gradient of stochastic objective functions.

### 2.3. Experimental Setup

In this experimental study, we use a graphics processing unit (GPU) from NVIDIA and a processor with an Intel Core i6 architecture. In addition, the proposed model was trained using Python 3.8, Keras framework and other DL libraries, as shown in Table 2.

## 3. Results

This section describes the proposed CNN-based model performance based on key performance metrics (Accuracy, Recall, Precision, F1-score) and key metrics (such confusion matrix, ROC, and time complexity (ms)) in addition to more deeply explained comparative studies.

### 3.1. Performance Metrics

In order to evaluate the performance of a classifier, it is necessary to differentiate between four distinct types that have been assigned to the target category: true positives, false positives, true negatives, and false negatives.

- TP: These are the positive values that have been accurately predicted, which indicates that the value of the real class is yes and that the value of the predicted class is also yes.
- TN: These are the negative values that have been successfully predicted, which indicates that the value of the real class is “no,” and that the value of the predicted class is also “no.”
- Both false positives and false negatives can arise in situations where the actual class does not match the class that was expected.
- FP: This situation occurs when the actual class is not yes but yes for the predicted class.
- FN: This situation occurs when the actual class is yes but the projected class is no.
- With the help of this matrix, it is easier to understand why the categorization model generates inaccurate predictions. This gives you an idea of the mistakes that are being made, as well as the categories of errors.

The following are the evaluation metrics that we used:

(1) $$Acc\;\left(\%\right)=\frac{TP+TN}{TP+TN+FP+FN}$$

(2) $$Prec\;\left(\%\right)=\frac{TP}{TP+FP}$$

(3) $$Recall\;\left(\%\right)=\frac{TPos}{TP+FN}$$

(4) $$F1-Score\;\left(\%\right)=2\times (\frac{Recall\times Prec}{Recall+Prec})$$

### 3.2. Confusion Matrix

**Figure 6:**
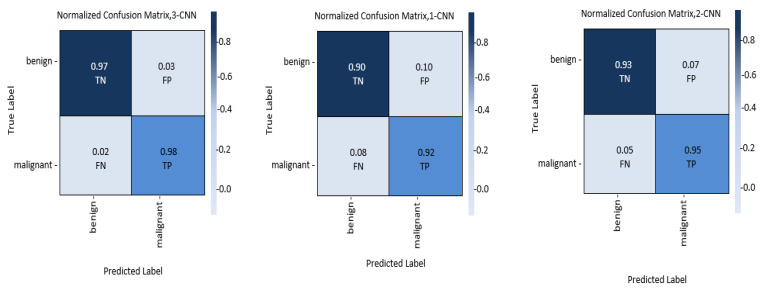
The retrieved confusion matrices of the introduced models (1-CNN, 2-CNN, and 3-CNN) for the diagnosis of BC histology.

Confusion matrix is implemented for displaying the outcome of the classifier model. A thorough experiment of the confusion matrix reveals that the fusion of 2-CNN and 3-CNN correctly recognizes the classes. The confusion matrix for all three models is presented in Figure 6, which shows that the proposed approach RNN-BiLSTM accurately detects the classes and performs better than the other two methods; i.e., RNN-LSTM and RNN-GRU.

### 3.3. ROC

**Figure 7:**
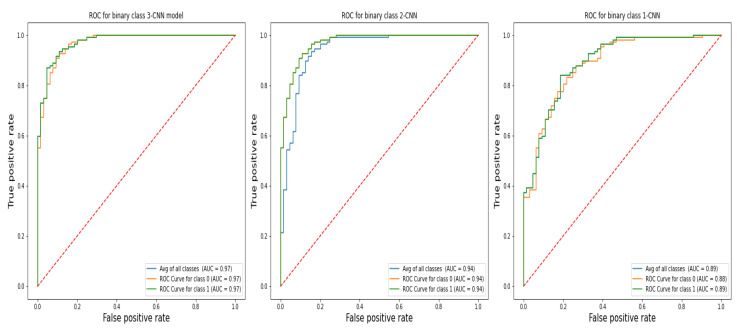
The retrieved AUC-ROC curves of the introduced models (1-CNN, 2-CNN, and 3-CNN) model for the diagnosis of BC detection and histology.

Receiver-operating characteristic, often known as the sensitivity-to-specificity ratio, is an experimental method used to evaluate the efficiency of diagnostic tests. Figure 7 illustrates the ROC of the proposed 1-CNN, 2-CNN, and 3-CNN models for the binary class recognition problem of BC diagnosis. In comparison of different CNN models, the ROC results show that the 3-CNN model successfully classifies malignant and benign cancer.

### 3.4. Accuracy, Recall, Precision, and F1-Score

**Figure 8:**
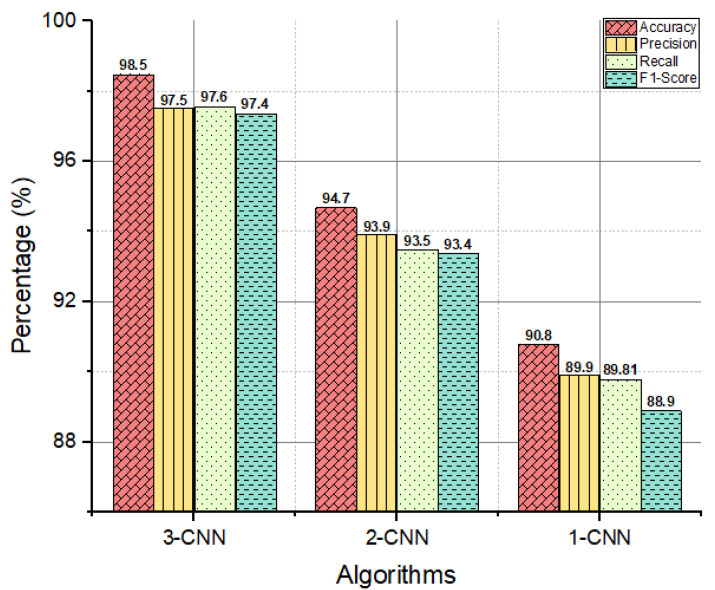
The retrieved accuracies, recalls, precisions, and f1-scores of the introduced models (1-CNN, 2-CNN, and 3-CNN) for the diagnosis of BC histology.

In this study, three models of CNN (1-CNN, 2-CNN, and 3-CNN) have been introduced to compare how well they classify cancer images. There are always two classes in a model, labeling 0 (benign) and 1 (malignant). The performance and effectiveness of a classifier can be evaluated based on its accuracy. It demonstrates the number of samples for which the model can adequately account for their characteristics. Figure 8 illustrates how accurate our proposed fusion CNN models are (i.e., 1-CNN, 2-CNN, and 3-CNN approaches). The accuracy, precision, recall, and f1-scores of the 1-CNN model were respectively 90.90%, 89.90%, 89.80%, and 90.20% while those of the 2-CNN model were 94.90%, 93.90%, 93.80%, and 93.20%, respectively. For the 3-CNN model, they were respectively 97.90%, 96.90%, 96.80% and 97.20%. The fusion 3-CNN model had the 3% best accuracy, precision, recall, and f1-scores than the 1-CNN and 2-CNN models, while the 1-CNN model had a poorer performance for BC detection and classification.

### 3.5. MCC Analysis

**Figure 9:**
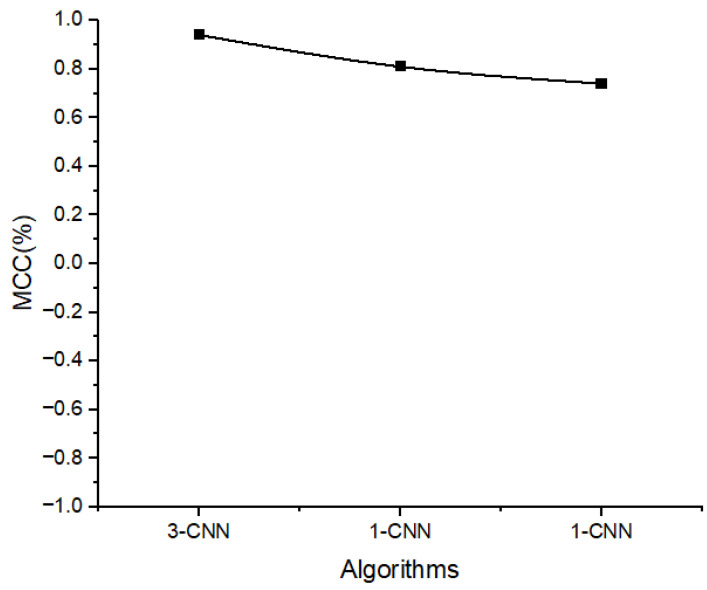
The retrieved MCC values of the introduced models (1-CNN, 2-CNN, and 3-CNN) for the diagnosis of BC histology.

The Matthews correlation coefficient (MCC) represents the coefficient ratio between TP and TN. The value of MCC is equal to one when the classifier is perfect (FP = FN = 0), which indicates that there is a perfect positive correlation. On the other hand, when the classifier constantly makes an incorrect classification (TP = TN = 0), we obtain a value of −1, which represents a perfect negative. In this study, the MCC value of the 3-CNN model is close to 1, which showed a perfect positive correlation as compared to the 1-CNN and 2-CNN models, as shown in Figure 9.

### 3.6. Time Complexity

**Figure 10:**
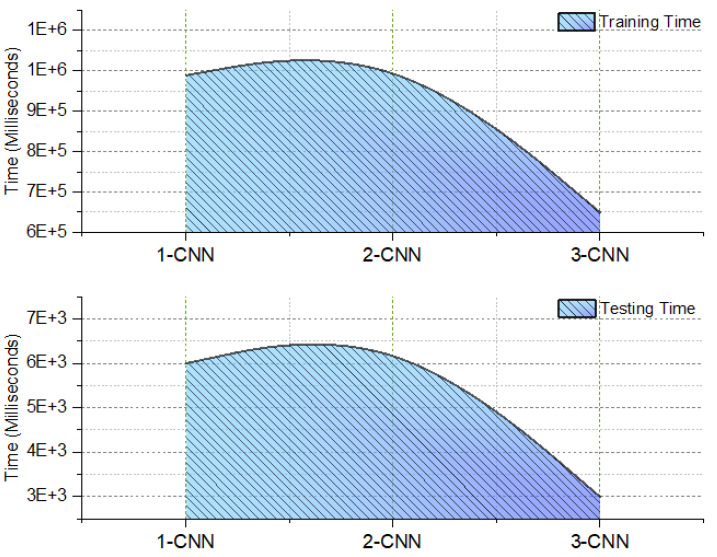
Time complexity (milliseconds) of the introduced diagnosing models (1-CNN, 2-CNN, and 3-CNN) for BC histology.

This section provides the time complexity of the various CNN model combinations, depending on the amount of time required for testing as well as training for classification of breast histology into benign and malignant tissues. The time complexity gives an indication of how effective the 3-CNN model is, which means that it requires a shorter amount of time for training and testing for BC detection, as shown in Figure 10.

## 4. Discussion

This study proposed different combinations of the CNN model (a simple convolutional neural network (CNN) model), as well as the fusion of two CNNs (also known as 2-CNN) and three CNNs (3-CNN) for BC detection. The features were extracted based on the CNN model. The proposed approach might be broken down into two primary categories: pre-processing (picture scaling and labelling), segmentation of the BC image, and the proposed CNN fusion model. In the data augmentation process, the number of images of malignant cancer was increased to improve the classification of the CNN model. We created and evaluated different fusion models that were trained and used to differentiate between benign and malignant classes.

The results of the experiments were derived from an analysis of our contribution to determine how the primary parameters affect the classification accuracy, specificity, and sensitivity; area under the receiver operating characteristic curve (ROC); and time complexity. In addition, the fusion of the CNN model is compared based on key performance metrics (i.e., accuracy (%), precision (%), recall (%), f1-score (%, ROC, confusion matrix), MCC, and computational time (ms)).The results, as shown in Figure 6, Figure 7 and Figure 8 reveal that when the fusion increased, the classification performance increased too; this means that the 3-CNN model performed better than the 2-CNN and 1-CNN models, in less computational time too.

Using the PCam database, we compared the accuracy of the introduced method to that of the state-of-the-art classification frameworks for BC histology. Because of this, we were able to gauge the significance of the hybrid-CNN method. Table 3 shows the differences between the proposed approach and the current SOTA methods. The AUC has been used as a performance metric for the comparison. Kandel and Castelli [56] have investigated how block-wise fine-tuning affected the performance of VGG16, VGG19, and InceptionV3, three popular Pretrained CNNs. The primary goal of their research is to determine if fine-tuning is an appropriate method for examining the PatchCamelyon histopathology dataset. In their study, they employed a batch size of 64 and three different learning rates of $10^{-3}$, $10^{-4}$, and $10^{-5}$. For the VGG16, VGG19, and InceptionV3, the achieved AUC values were 0.94, 0.93, and 0.92, respectively. Lower values for the learning rate have been reported to yield the best results when training those pretrained CNNs. An ensemble model for the classification of the PCam histopathology dataset has been presented by Kassani et al. [57] that integrates three pre-trained CNNs (VGG19, MobileNet, and DenseNet). The feature representation and extraction processes have both made use of the ensemble model. The classification operation is executed by feeding the retrieved features into a multi-layer perceptron classifier. Normalization, data augmentation, and fine-tuning are among the various pre-processing and CNN tuning approaches used to train the model. Using PatchCamelyon histology datasets, the multi-model ensemble method achieves higher accuracy (94.64%) in its predictions compared to single classifiers and machine learning algorithms. The dense-attention network (DAN) is a method that has been presented for the classification of malignant patches by Liu et al. [58]. This method involves the attention mechanism being further enhanced in order to include prior knowledge about the surrounding tissue. In addition to this, the usefulness of data augmentation in the Inference stage has been verified even further. Using the PatchCamelyon dataset, where images with cancerous tissue in the middle are classified positive while those from the surrounding regions will not affect the label, the proposed approach is tested and evaluated. With the PCam dataset, they achieved an AUC of 0.976 successfully. An ensemble-based paradigm for the classification of lymph node metastases has been proposed by Rane et al. [59] DenseNet201, InceptionV3, and ResNeXt-50 are some of the pre-trained CNN models that are included in the aforementioned ensemble framework proposal. An attention fusion network has been implemented inside the framework that has been proposed in order to aggregate the predictions that have been generated using the different models. The introduced model obtained an AUC-ROC score of 0.9816 when used with the PCam benchmark dataset. Bonne [60] conducted a study in which she investigated various network architectures of CNN for the purpose of detecting breast cancer based on microscopic images of sentinel lymph node tissue. With the PatchCamelyon dataset of lymph node tissue, convolutional models of increasing depth are trained and assessed. It has been determined how much of an influence transfer learning, data augmentation, and fine-tuning of hyperparameters have. They found that increasing the depth of CNN had the greatest impact on performance, but data augmentation and transfer learning did not. With the help of the InceptionV3, they were able to get a value of 0.95 for the AUC performance parameter. Lafarge et al. [61] have proposed a new histopathological patch-classification model called P4M-DenseNet. This model improved upon the competitive standard CNN by mandating that rotation and reflection equivariance be maintained. The densely linked convolutional network serves as the foundation for the patch-classification model that was suggested. The performance of the P4M-DenseNet model was measured and analyzed using the PCam benchmarking dataset. The area under the curve (AUC) that was achieved for the classification of the microscopic images that were included in the PCam dataset was 96.3. By comparing the findings discussed earlier in this section to our proposed framework, we found the following: the performance level attained by the newly designed framework is higher than that achieved by Kandel and Castelli [56], Kassani et al. [57], Liu et al. [58], Bonnet [60], Rane et al. [59], and Lafarge [61].

While the proposed deep learning method showed impressive performance in distinguishing between benign and malignant breast tissue in histological images, it is handicapped in terms of the interpretability and explainability. Real-world applications, such as medical imaging, have gained great benefits from the use of deep neural networks. From a research perspective, however, black-box AI systems that lack or have limited explainability, and interpretability remain obstacles. Because of their non-linear and complex nature, deep neural networks lack interpretability compared to simpler and self-explaining models (such as linear regression). Black-box solutions in medical imaging and other fields are not easily deployed for mission-critical decision making, despite the deeper models’ ability to recognize and model complicated patterns and enable much improved performance. To address this issue, explainable artificial intelligence (XAI) techniques may offer explanations for the DL model black box, judgments that humans can understand. By assessing and reviewing individual predictions through analyzing their reasons, XAI approaches may assist physicians in further evaluating their models beyond typical performance metrics [62]. Furthermore, these techniques may expose biases in the training data, multiple labels, multi-input, and other inaccurate or fictitious correlations discovered using a model. Other forms of XAI include the technique known as Grad-CAM, which stands for Gradient-weighted Class Activation Mapping [63]. This method was first presented by Selvaraju et al. [64] in their study. In order to provide a post hoc local explanation, Grad-CAM can work with any sort of CNN, whereas CAM requires global average pooling in order to function properly. In addition to this, the authors presented guided Grad-CAM, which may be thought of as an element-wise multiplication between guided backpropagation and Grad-CAM. In the field of medical image analysis, both Grad-CAM and Guided Grad-CAM have been utilized. For example, Ji [65] utilized Grad-CAM to show which portions of the lymph node segments could be used as a classifier for the histology of metastatic tissue, and Kowsari et al. [66] used it to detect small bowel enteropathies on histopathology. Both groups of researchers found that Grad-CAM was an effective tool.

In order to improve the explainability of AI models, many applications of Scene graphs are being explored in the healthcare industry. They annotate the data, arrange it, and show it in a comprehensible way. Moreover, they add semantic labels to the healthcare datasets. An XAI model takes advantage of the structure of Scene graphs, which is built on subject–predicate–object relationships, in order to describe concepts and the connections between them [67]. It is possible to apply multi-modal machine translation methods in order to train a shared multilingual visual-semantic embedding space [68]. This may be useful for the purpose of improving the CNN models’ existing multi-modal capabilities. Furthermore, the transfer learning strategy for temporal activity detection [69] might be helpful for boosting the performance of CNN models for temporal BC histological images. This is because transfer learning is used to identify patterns of activity over time.

Another limitation of deep learning models in the diagnosis of BC histopathology is the requirement of extensive annotated datasets provided by physicians or other specialists in order to train the model. These datasets are needed to train the model. Zero-Shot Learning, also known as ZSL, is a relatively new method that has recently been developed to address this issue [70]. ZSL is presented for the purpose of having little human interaction by relying exclusively on previously known or trained concepts in addition to auxiliary information that is now available. Generative adversarial networks have made amazing strides in ZSL in recent years, which has led to some exciting new developments (GAN). An explosion of GAN designs has been constructed by human experts through trial-and-error testing in an effort to compensate for the dearth of training data available in ZSL [71].

## 5. Conclusions

In this study, we proposed a total of three models: the 1-CNN fusion of the two CNNs (2-CNN) and three CNNs (3-CNN) for BC pathological image classification. Initially, different pre-processing methods were applied in order to obtain the highest possible classification performance. Additionally, the experimental study was conducted by using the well-known Kaggle dataset (PCam). The results of the experiments indicated that the performance metrics of accuracy, recall, precision, and F1-score of the 1-CNN model were 90.10%, 89.90%, 89.80%, and 89.90%, respectively; the values for 2-CNN model were 94.7%, 93.9%, 93.4%, and 93.4, respectively, while those for the 3-CNN model were 97.90%, 97.5%, 97.60%, and 97.40%, respectively. The fusion 3-CNN model accurately classifies BC pathological images into benign and malignant cancerous lesions. Furthermore, the fusion 3-CNN model overcomes the issue of incorrect classification and time-complexity problem and has significantly better performance than the fusion 2-CNN and 1-CNN models, respectively.

In future studies, the proposed CNN fusion model will be expanded to breast tissue detection and classification in order to help medical professionals better identify diseases. The use of XAI approaches may make it possible to provide explanations for the introduced CNN model, sometimes known as the black box, and evaluations that humans can understand. Further objectives would be to test our approach using a more challenging breast cancer images dataset, which would enable us to demonstrate the model’s durability. We want to extend this method to other areas of healthcare, particularly those in which it has the potential to benefit the whole biomedical community.

## Figures and Tables

**Table 1:** An overview of the State of The Art, SOTA, methods and their limitations for the automatic diagnosis of the abnormalities in breast lesions’ histopathology.

Author	Approaches	Dataset	Strong Points	Weak Points
Bayramoglu et al. [48]	CNNs	BreaKHis dataset	Malignancy prediction using a single-task CNN and simultaneous prediction of malignancy and image magnification level using a multi-task CNN are the two architectures proposed.	They were unable to achieve better performance than the state-of-the-art results.
Arajo et al. [49]	CNN CNN + SVM	Breast histology classification challenge H&E stain samples from Bioimaging 2015.	They have completed image-wise classification into four medically relevant classifications, as opposed to the two classes used via earlier methods: (i) normal tissue, (ii) benign lesion, (iii) in situ carcinoma, and (iv) invasive carcinoma.	Patch classification performance may be decreased if some training/testing patches lack significant information.
Aresta et al. [50]	CNN-based algorithms	BACH dataset from the ICIAR2018 competition	Effective methods for the Automatic classification systems using hematoxylin–eosin-stained histopathology images	The study findings are not yet satisfactory for clinical evaluation.
Ahmed et al. [51]	Transfer learning (AlexNet/ResNet/GoogLeNet)	Breast Histology dataset	The implemented strategy has brought about an accuracy rate of 85% in the instance of ResNet, which is the highest among others.	The design that was presented can be improved to allow better diagnosing accuracy.
Vang et al. [52]	Google Inception V3 Network	BACH dataset from the ICIAR2018 competition	The introduced framework has resulted in a 12.5% enhancement over the SOTA models	The findings of the study do not yet meet the criteria necessary for clinical assessment.
Rakhlin et al. [53].	VGG-16+ Gradient boosted trees classifier	BACH dataset from the ICIAR2018 competition	They reported an accuracy rate of 87.2% when doing the 4-class categorization challenge. They reported an accuracy of 93.8% when asked to do a 2-class classification test to identify carcinomas.	Lack of comparison with other pretrained CNNs
Awan et al. [54]	ResNet	BACH dataset from the ICIAR2018 competition	A novel framework has been presented. ResNet is introduced for feature extraction, PCA for feature reduction, SVM for patch classification, and a majority voting classifier for overall input image classification.	When compared to the accuracy retrieved using SOTA models, the achieved accuracy of 90% is lower.
Proposed method	Fusion of CNNs (1-CNN, 2-CNN, 3-CNN)	PCam dataset	The performance of the suggested model, which utilizes hybrid CNNs, is shown to be significantly enhanced when it comes to correctly and precisely identifying abnormalities in breast histology, which suggests that its application in this field should be expanded.	

**Table 2:** Experimental setup.

GPU	1060 6 GB, Nvidia GeForce
RAM	16 GB
Languages	Python 3.7
CPU	Core-i6, 6th Generation, and 2.80 GHz processor
Operating system	Windows 64 bit

**Table 3:** Comparing the attained classification performance to the state-of-the-art for the diagnosis of BC histology using the PCam dataset.

Author	Approach	AUC	Accuracy
Kandel and Castelli [56]	Transfer learning	0.94 (VGG16) 0.93 (VGG19) 0.92 (InceptionV3)	-
Kassani et al. [57]	Ensemble model (Pre-trained CNNs + Multi-layer perceptron classifier)	-	94.64%
Liu et al. [58]	Dense-Attention Network	0.976	-
Bonnet [60]	CNN, InceptionV3	0.95	-
Rane et al. [59]	Ensemble framework (DenseNet201, InceptionV3 and ResNeXt-50)	0.9816	-
Lafarge [61]	P4M-DenseNet	96.3	89.8
Proposed model	1-CNN, 2-CNN, and 3-CNN based fusion model	0.89, 0.94, and 0.97	90%, 94%, and 98%

## Data Availability

Data will be provided upon request.

## Abbreviations

The following are the abbreviation used.

DL	Deep learning
AI	Artificial intelligence
ML	Machine learning
GPU	Graphics processing unit
RNN	Recurrent neural networks
LSTM	Long short-term memory
CNN	Convolutional neural network
